# Correction: Guo et al. Shikonin as a WT1 Inhibitor Promotes Promyeloid Leukemia Cell Differentiation. *Molecules* 2022, *27*, 8264

**DOI:** 10.3390/molecules31030411

**Published:** 2026-01-26

**Authors:** Zhenzhen Guo, Luyao Sun, Haojie Xia, Shibin Tian, Mengyue Liu, Jiejie Hou, Jiahuan Li, Haihong Lin, Gangjun Du

**Affiliations:** 1 Henan Province Engineering Research Center of High Value Utilization to Natural Medical Resource in Yellow River Basin, Pharmaceutical College, Henan University, Kaifeng 475004, China; 2 School of Pharmacy and Chemical Engineering, Zhengzhou University of Industry Technology, Xinzheng 451100, China

Following publication of the original article [1], the authors noticed an error in Figure 4E (β-actin).

During the experiment, the same protein imaging system was used in different experiments, and the β-actin bands were named the same, which led to errors in data storage and subsequent picture presentation (an error in Figure 4E (β-actin)). After rechecking, it was found that β-actin protein bands were used incorrectly. The image with the error and the corrected image are shown in the following figure. We sincerely apologize for this mistake.

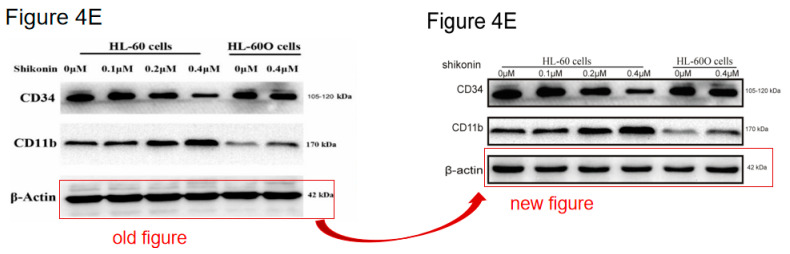



We sincerely apologize for any confusion this error may have caused and appreciate the opportunity to rectify it. We thank the editors and readers for their understanding. And these corrections do not impact the overall findings and conclusions of the paper. We would like to assure readers of the interpretations and validity of the research.

The authors state that the scientific conclusions are unaffected. This correction was approved by the Academic Editor. The original publication has also been updated.
